# Key considerations for an inclusive framework for youth with disabilities in post-apartheid South Africa

**DOI:** 10.4102/ajod.v11i0.954

**Published:** 2022-11-11

**Authors:** Marlene F. le Roux

**Affiliations:** 1 Centre for disability and rehabilitation Studies and AfriNEAD, Department of Global Health, Stellenbosch University, Cape Town, South Africa

**Keywords:** inclusive development, disability, youth development, empowerment, humanity, dignity

## Abstract

**Background:**

The South African Constitution asserts that persons with disabilities must have equal access to opportunities in society; however, the realisation of this mandate has remained a challenge. There is a need to create contextually relevant, inclusive structures that support equal access to opportunities for persons with disabilities in society.

**Objectives:**

This article reflects on and highlights key considerations for an inclusive framework that facilitates access to opportunities for youth with disabilities in South Africa, which emanated from a study that explored how ongoing interaction with the performing arts can facilitate social and economic inclusion of youths with disabilities.

**Method:**

The study adopted a qualitative research approach, using critical ethnography. Primary data were obtained from three focus groups with a total of 20 youth with disabilities who have attended performance events, as well as an in-depth interview with a disabled performer.

**Results:**

The facilitation of access to equal opportunities for youth with disabilities must occur at a multidimensional level, involving both personal and systemic changes and levels of support. Complex barriers linked to the apartheid legacy also exist, some of which include access to resources and reduced self-determination, whilst positive factors such as internal resilience and skills development function as promising predictors of inclusion.

**Conclusion:**

Contextually relevant, disability-inclusive structures in South Africa must confront and address how youths with disabilities are uniquely impacted in present times by South Africa’s history.

**Contribution:**

The voices of youths with disabilities make a key contribution as their experiences must inform these inclusive structures which have the potential to enhance access to equal opportunities for them at both personal and systemic levels.

## Introduction

The United Nations Sustainable Development Goals (SDG 2030) and the United Nations Convention on the Rights of Persons with Disabilities (UNCRPD 2006) advocate for the inclusion of persons with disabilities in all spheres of society (Jolley et al. 2018). The South African Constitution asserts that persons with disabilities must have equal access to education, employment, basic services and reasonable accommodation; however, relatively marginal changes have been made within societal structures to effectively provide equal access to persons with disabilities (Ngwena & Pretorius 2012).

The understanding of disability in this article is aligned to the UN Convention on the Rights of Persons with Disabilities and the International Classification of Functioning (ICF) (WHO 2001) definition of disability, which steps away from the impairment-focused deficit model of disability. Therefore, I define disability as:

[*T*]he loss or elimination of opportunities to take part in the life of the community, equitably with others, which is encountered by persons having physical, sensory, psychological, developmental, learning, neurological or other impairments, which may be permanent, temporary or episodic in nature, thereby causing activity limitations and participation restrictions within the mainstream society. (Stats SA 2011:13)

The given definition clearly heralds the need to address this loss of opportunities for people with disabilities (PWDs).

Historically, PWD have been marginalised globally and are currently still experiencing marginalisation despite various international and governmental policies and treaties that advocate for their rights (Sa’ar & Aratan-Bergman 2016). They are prevented from accessing healthcare, education and economic opportunities on an equal basis with other members of society (Albert & Hurst 2004). They are often viewed through a medical deficit lens that identifies whatever impairment they have as the reason PWD are excluded from accessing equal opportunities and, in some instances, they are even perceived as being a negative influence on society (Dossa 2008).

The world report on disability (2011) stated that the African region holds a higher number of persons with disabilities that are moderate to severe, most of whom are in the younger generation, under 60 years of age. Adding to this complexity is the high prevalence of poverty and governments’ incapacity to adequately address these challenges in a manner that facilitates access to opportunities and the inclusion of youth with disabilities within society, which often leads to further marginalisation of this vulnerable group. There is a need to create inclusive structures that support equal access to opportunities for youth with disabilities in society. This article presents key considerations for creating an inclusive framework within societal structures by facilitating access to performing arts opportunities for youth with disabilities in South Africa. This has the potential to support personal growth and social inclusion of youth with disabilities.

Within South Africa, historical divisions along racial and gendered lines created major social divisions during the apartheid era. People with disabilities were further oppressed and had limited access to public spaces and opportunities (Ngwena & Pretorius 2012). The patterns of inequality created during apartheid contributed to structural and systemic barriers to inclusion for PWD, which are still experienced to date.

Persons with disabilities currently make up more than 7.5% of the nation’s population but have seen little change in the area of access, for instance, to opportunities for economic and social participation (Bam & Ronnie 2020; Ebrahim et al. 2020; Ned & Lorenzo 2016). Many of this vulnerable sector live in urban townships or rural areas where services are especially difficult to access and, despite the progressive element of the Constitution, have been left behind (Lorenzo 2008; Ned & Lorenzo 2016). This includes youths with disabilities.

Young people under the age of 35 make up 66% of the South African population (Hanass-Hancock et al. 2018). These young people generally encounter a lot of structural and attitudinal challenges within society and are even more challenged by societal and systemic barriers (Hanass-Hancock et al. 2018). Youth with disabilities require adequate care, medical equipment and rehabilitation, accessible buildings and transport, as well as emotional support from family members and care workers (Le Roux 2018). These factors are single or synergistic satisfiers of human needs for social and economic development and would facilitate better inclusion of youths with disabilities in our society (Le Roux 2018).

The National Youth Policy (SA, WYP 2020–2030) outlines a youth development strategy for South Africa (Department of Women, Youth and Persons with Disabilities 2020), creating an avenue for youth with disabilities to be represented and included in development opportunities through the Ministry for Rural Development and Land Reform. This is equally aimed at enhancing the use of local resources to facilitate inclusive opportunities for youth with disabilities (Lorenzo & Motau 2014). Artscape and the many programmes it offers for young people and youths with disabilities, for instance, is one such local resource that can be used to facilitate the inclusion of youths with disabilities (Le Roux 2018). The present situation highlighted here has informed this study. This article reports on participant narratives on key aspects of inclusion which can facilitate access to opportunities and social inclusion of youth with disabilities. This emerged from a study that explored how interaction with the performing arts may facilitate the social and economic inclusion of youth with disabilities (Le Roux 2018) by fleshing out their marginalised position within the context of a post-apartheid South African (Le Roux 2018).

## Theoretical framework and methodology of the study

This section briefly explains the theoretical framework, methodology and research design followed in the collection, organisation and analysis of data and positions the researcher within the study. There are four theoretical frameworks that guided the study: feminist ethics of care (Gouws & Van Zyl 2015; Kittay 2011; Yuval-Davis 2006), which advocates for a collective approach to address injustices based on gender; models of disability (Langtry 2010; Moyne 2012) (medical, charity, social, rights-based and affirmative); assisted exploration of the definitions of disability holistically; intersectionality (Crenshaw 1989; Yuval-Davis 2006), which supported the identification of the multiple intersecting systems of oppression emanating from my participants’ experiences, history and context; and human scale development (Max-Neef 1992), which asserts that all fundamental human needs are culturally and historically constant and that humans share a deep need to satisfy themselves (Le Roux 2018).

Research has shown that the lived experience of disability is mostly different for male and female people, and the researcher, as a female person with a disability, has lived experience of this difference. The gaze that is cast upon the female disabled body often breeds further marginalisation and sometimes even violence.

In addition, the study has an emancipatory element of opening up marginalised spaces and the discourses within those spaces. Therefore, as advocated by the feminist ethics of care and intersectionality, it became relevant to ensure that the voices of young women with disabilities were equally represented, to explore how these young women experienced disability and access within their own contexts. It was aimed at bringing out the female narratives and the intersecting locations of their lives to repudiate assertions and assumptions based on their gender and/or gender roles and to address the challenge of inadequate role models for women with disabilities. This emancipatory strategy was carried through in the methodology by ensuring representation across both men and women during recruitment and sampling.

The theories used in this study – intersectionality, ubuntu, feminist ethics of care and the Max-Neef theory of needs satisfaction – all validate the given facts. They include empathetic understanding and care, interconnectedness and inter-relatedness, participation, solidarity, community and reciprocal commitment to each other’s well-being. These are the foundational concepts of this study, as youth with disabilities seek a space of recognition of shared humanity, caring social relationships and equal participation on all levels of beingness.

### Study context, methodology and research design

Study participants were recruited from the Cape Flats, which is an area located between the City Centre of Cape Town and Cape Town International Airport. The Cape Flats consists of predominantly black townships, ‘coloured’ (formerly Cape coloured, a person of mixed European [‘white’] and African [‘black’] or Asian ancestry, as officially defined by the South African government from 1950 to 1991) ghettoes and shantytowns. This area was the place to which most black people were relocated, and migrant labourers also settled there after other areas were declared ‘whites only’ during apartheid and the *Group Areas Act* was passed. This area was commonly referred to as ‘the dumping ground’, which is characterised by high levels of poverty and, historically, the marginalisation of youth (South African History Online 2021).

### Methodology

A qualitative research approach was adopted using a critical ethnographic research design. In essence, critical ethnography explores the culture, community and daily circumstances of participants, considering what is currently occurring and possibilities for the future (Thomas 1993). Boylorn and Orbe (2014:15) asserted that critical ethnographers are interested in the ‘politics of positionality’, where researchers expose their own privileges in addition to their marginalisations and ‘take responsibility for [*their*] subjective lenses through reflexivity’. Critical ethnographers aim to explore, understand and interpret the dynamics within a group, identifying past and present patterns of injustice that may have consequences for current life experiences. Finlay (2006) agrees with Richardson’s (2002) evaluation of ethnographic research. Both authors advocate offering both ‘scientific’ as well as ‘literary’ aspects of research and using diverse ways to present ethnographic research. Both argue that reflection on the personal, emotive narrative is as important to evoke as the so-called positivist, typical rendition of what scientific knowledge is or should be. Thus, the researcher is located as a potential influence within the research process and must therefore explore any subjective bias they may have. As a black woman with a disability, and as someone from a historically marginalised community, I am aware of my closeness to the data. Therefore, reflexivity is a valuable tool for me to evoke the literary aspect of my research and draw from critical ethnography and my positionality in this study, to confront previous practices of discrimination and to address this by ensuring an authentic representation of the voice of my study participants that aligns to their subjective experiences of access to opportunities as youth with disabilities (Le Roux 2018). Therefore, I position myself in the next section.

### Positioning the researcher

I am a woman of mixed race from a rural town, Wellington, South Africa. I contracted polio when I was three months old. This is because I grew up in a segregated community under apartheid, hence the white clinic refused to administer the polio drops to me as a mixed-race person. This is because mixed-race people could not receive vaccination in any clinic segregated for white people only. My family was poor and struggled socio-economically; however, plenty of love, care and support was given to us as we grew up. This love and care, however, could not protect me from the systemic discrimination and marginalisation I experienced growing up. As a 54-year-old woman today, I have first-hand knowledge of the impact of these deficit systems on the growth and development of young people. I had neither access to a special needs school nor to accessible transportation, as there was no public transportation for persons of mixed race in the rural town of Wellington. For example, when considering university in the 1980s, I enthusiastically sent an application to the music faculty of one of the top higher education institutions in the country, with a very excellent art programme to be an opera singer, only to be turned down because of my impairment; they stated that they had no space for a disabled opera singer. This is one reason that the theory of intersectionality is important to me, and even more so as it has truly played out in my own life.

Despite this setback, I was taught to push and not give up by my family and to trust in my own will and capacity to succeed. So I learnt to improve my understanding of people and the arts, especially how to contribute to resilience for youth – with and without disabilities – in my community and at all levels of society, encouraging them to pursue their dreams. My experiences have further strengthened my understanding of the key issues of access that persons with disabilities have to face. I align with Shah’s (2006:208) assertion that young people are ‘active social agents, able to articulate their own experiences and express their views’. This has informed the work I do with youths with disabilities. Therefore, in this study, whilst narrating my marginalisation, I equally acknowledge my methodological privilege as a disabled researcher. I am positioned as ‘a research tool’ to consciously elicit the narratives and experiences of inclusion by these young PWDs (Shah 2006:218). This supported me in identifying current barriers inherited from the history of systemic oppression and collectively identifying sustainable, contextual ways to address them and to facilitate inclusion.

### Study population

The study population are youth with disabilities who have attended at least one or more events at the Artscape Theatre. This sample also includes youth with disabilities from historically disadvantaged communities in Cape Town (Cape Flats area). Youth comprise young people between the ages of 18 and 35 years of age (*South Africa Youth Commission Act* 1996), so this guided the categorisation of youths.

### Participant sampling, inclusion criteria

A purposive sampling strategy was used for the study. All participants were youth, aged between 18 and 35 years, as defined by *South Africa’s Youth Commission Act* (1996). They self-identified as male or female and had self-described sensory, mobility, mental or psychosocial disabilities. They are bilingual, as the researcher needed to conduct the interviews in both English and the participants’ spoken local language.

### Recruitment strategy

The recruitment of participants occurred across three different settings and there were three focus groups and one interview held, as described here.

The Cape Flats comprises poor, disadvantaged, black and mixed-race communities in the Western Cape province, South Africa – I contacted disability organisations because as a person with a disability, I am familiar with these organisations and their work with youth with disabilities. The organisations approached are Woman’s Achievement Network for People with Disabilities (WAND) – they work with young women with disabilities; UNMUTE – a professional dance company working with young PWD; Stigting vir Bemagtiging deur Afrikaans (SBA) – a cultural organisation that brings young PWD to the theatre; and attendees of the Unmute ArtsAbility festival.

These organisations then contacted their members in the Cape Flats area and informed them about the research. They gave my contact details to people who were interested. Some had access to phones and called or sent a message to me. I then called them back to explain further. Next, because some of the interested youth did not have phones, I organised transport with the help of these nongovernmental organisations (NGOs) to collect all the youths who were interested in participating and bring them to a venue where we met and I clarified and explained more about the research, answered all their questions and sought their consent, which they gave by signing the consent forms. Two additional groups from training institutions were also identified and invited because students from these schools often attend performances at Artscape. The consent form was sent to the principal, who gave it to the learners, and those who were interested contacted me.

Therefore, the three groups and one individual recruited were as follows:

- Six learners from the Tertiary Training College for the Deaf attending a theatre performance as audience members.
- Seven Grade 12 learners from a Learners with Special Education Needs (LSEN) school who attended a production of an Afrikaans Setwork 3 and were facilitated by the NGO SBA.
- Six disadvantaged community youth (individuals who have finished school and were currently at home and unemployed, recruited through NGOs as discussed here).
- One individual interview which was conducted with a performer with a disability at Artscape.

Focus group sessions were held with the three groups. The focus group discussion was specifically chosen over individual interviews because of the vulnerability of these young PWDs. Being in a group meant that they could support each other to speak and share stories; in this way, peer support was encouraged. In addition, because of the isolation they often experience as a result of their disability, they had few social interactions. Therefore, being in a focus group meant that they could socialise whilst participating in the research.

Data were also collected through a document analysis from the Artscape Universal Access and Design Task Team’s database and two in-depth individual interviews with the black disabled female performer. In addition, three focus groups were conducted with a total of 19 participants from the schools and the youth with disabilities from disadvantaged communities. The researcher’s reflective journaling of the research process was used as secondary data to support analysis.

### Data management and analysis

All data transcripts and field notes used in this research are not in the public domain. All data were stored by the researcher on a password-protected computer file, and it will be stored for five years as stipulated by the University of Cape Town.

Data analysis began with the transcription of all digitally recorded data. Creswell (2007) suggested that in reading through data and gaining familiarity, one can start the process of understanding it. Thematic analysis, as described by Bowen (2009) and Braun and Clarke (2006), was used to identify recurring themes and patterns in the focus group interactions, specifically focusing on a critical ethnographic concern with patterns of social injustice. Braun and Clarke (2006:78) viewed thematic analysis as a flexible tool for research, ‘which can potentially provide a rich and detailed, yet complex, account of data’.

Thematic analysis enables researchers to make sense of data in accordance with their specific focus and within a broader methodological framework (Braun & Clarke 2006). The researcher established a manual coding system to identify recurring themes and patterns emerging from the data and effectively categorise responses (Bowen 2009; Braun & Clarke 2006; Marshall & Rossman 2006). Any additional comments were included as a means to contextualise focus group transcripts. Review and constant comparison enabled the researcher to place codes into categories and subcategories and the verification of themes and categories was carried out up to the point where saturation was reached. Pseudonyms were used to identify participants and their responses, ensuring that confidentiality and privacy remained uncompromised.

The documents’ review outcomes were analysed using the Statistical Package for Social Sciences (SPSS version 24, IBM Corp. 2016). A detailed discussion on the study methodology has been published in a separate article (Le Roux, Kathard & Lorenzo 2021).

### Ethical considerations

Ethical approval to conduct the study was obtained from the Human Research Ethics Committee of the Faculty of Health Sciences, University of Cape Town (clearance number: HREC 6001/2016). Participants were informed about the nature of the study and that it is a low-risk study but any risk that arises will be minimised. They were handed informative handouts and given opportunities to ask questions for clarification through question-and-answer sessions before giving consent. They were informed that could withdraw at any time without any negative consequences. Data were anonymised to protect the identity of study participants and enhance confidentiality. All participants were treated with dignity and respect (Le Roux 2018).

The following section presents participant narratives of inclusion, what they identify as key factors that they believe facilitate their access to opportunities and the challenges they experience as young people for inclusion.

## Findings

### Interactive spaces and social connections

Participants identified the very important need to be able to fit into society as much as any other young person as a basic right they should be able to exercise. The capacity to go out and be socially included is a necessity and one which they believe begins with the creation of opportunities and having receptive attitudes. The participants from the Training Institution focus group also gave an example about how the arts can create spaces of interaction:

‘For me it’s a very fun and exciting space to be in because you interact with different people and you always live with new friends and stuff. And because I’m a person who likes people, I like to, I always make new friends but I’m never the one who’ll go to a person and be like them, but somehow people always find a way in finding me.’ (Chidera, student, theatre attendee, Focus Group [FG3], 2017:36)

‘So by creating such opportunities for young people, I think it does start changing the way we live and our societies. It starts giving young people hope and it makes them realise that they have the skills, they have the tools but it’s just a matter of working on them.’ (Samantha, community member, interview, March 2017)

‘Arts and culture – well, that space definitely does make it easier to transition into that space, because a lot of us who are confined within our homes or who don’t go out, it’s harder for them to transition into society as a whole. So arts and culture make you get used to the idea that there are people and you have to integrate yourself to society and it’s, as much as there is limit to the space but you can always be creative around because there are times, if you can’t get into a concert then we can sit outside and have our own thing, you know.’ (Abigail, student, theatre attendee, FG3, 2017:38)

### Positive identity and agency

These opportunities should be facilitated by consciously supporting youth with disabilities to grow their identity and sense of self. One participant stated that this positive sense of self helps to negate a history of marginalisation and contribute to a sense of agency for young people:

‘[*I*]f you sit and wait for funding to come to you, you are going to wait forever. I was placed in that position where I was forced to start creating opportunities for myself to be able to have work.’ (Yinka, student, theatre attendee, FG3, 2017:13)

Parental support was highlighted by participants as a critical factor that contributes to their confidence and agency. One participant from the focus group who frequently attends events and performances stated here that the values she carries were instilled by her parents, whilst another participant from the training institution felt inspired to support youth to respond to challenges and build their self-agency by creating an organisation:

‘The values that they instilled were always that you don’t wait for someone to hand something to you, you go out and you do something for yourself.’ (Samantha, community member, interview, 2017:8)

‘Then it kicked back home that I need to start an organisation for PWDs but mainly focus on the young people at school, because growing up with these challenges, they need to face the challenges that’s out there because accepting the fact that you are disabled, you can’t go there and you can’t do this because of all these stumbling blocks, it’s not gonna help, it’s just keeping you back.’ (Chidera, student, theatre attendee, FG3, 2017:36)

Participants believe that support systems, which contribute to agency-building for youth with disabilities, will motivate and inspire them to realise they can reach higher and aim for more, as exemplified by a deaf learner in high school. The school learners were all white people or mixed race. The deaf white learners were more affluent and clearly voiced their career aspirations:

‘My goal is to go and study next year – IT programming – and then I would like to be a speaker in Parliament and to work within the deaf community.’ (Howard, student, theatre attendee, FG2, March 2017:8)

Contrary to this, many of the mixed race and black learners were from disadvantaged, low socio-economic communities. They were hesitant to discuss their career options and some aspired to become skilled labourers, whilst others looked towards a career in the arts, although they still battled with a lack of confidence and fear:

A jewellery-maker, [*although*] my main thing I want to do is to build houses.’ (Brandon, student, theatre attendee, FG1, March 2017)

‘Okay, now I sing a lot. I go out to concerts, I perform in churches, I perform in all that other stuff but now I do less. I don’t know what happened, but I suddenly lost confidence and suddenly now I have so much fear that I never had when I was a teenager, but when it comes to music, I’ve got much confidence in it.’ (Blanche, student, theatre attendee, FG3, 2017:25)

On the other hand, some barriers to inclusion and access to opportunities were also identified by participants. Some of the challenges stemmed from personal challenges, and some were systemic and structural, but all impacted on their capacity to access opportunities in one way or another.

### Mobility challenges

The youth with disabilities who participated in the study identified certain challenges that were barriers to inclusion; public transportation was identified as a major challenge to accessing opportunities and inclusive spaces. The challenge was not only related to accessing transportation, but even getting to the taxi rank from their houses was often very challenging and strenuous due to the condition of the public roads within their environment:

‘The challenge is I can’t go alone to take the transport because of the roads, and also it’s not wheelchair friendly for me to be able to travel alone. I need to take a bus and Golden Arrow; sometimes they have an issue with me using the bus, then they want me to pay for the wheelchair and for myself as well.’ (Chidera, student, theatre attendee, FG3, 2017:19–21)

‘My mode of transportation is Dial-a-Ride, which … means that I have to book, always have bookings in advance. So if I want to go somewhere it has to be 7 days in advance for me to make the booking. So if there comes up an event tomorrow, I can’t attend because I haven’t made prior bookings to go to the event.’ (Dylan, student, theatre attendee, FG3, 2017:21)

### Access to resources

Another dimension that emerged from the findings of the study, based on participant narratives, pointed to the impact of South Africa’s history on youth with disabilities in terms of resources required for access. One alternative to problematic public transportation is privately owned transportation. Most of the white youth could access privately owned transportation, while black and mixed-race youth struggled to do so.

‘Currently we are busy building up my own car so that I will be able to drive here on my own next semester.’ (Koos, student, theatre attendee, FG2, 2017:7)

Participants also felt that the impact of this resource constraint even influenced their experience of inclusive spaces. This is because even when structures are inclusive, many people who manage these spaces lack disability sensitivity, and therefore often do not realise the need for additional accommodation for persons with disabilities.

‘People with disabilities aren’t being catered for; maybe you could afford those front row tickets, but because that isn’t accessible, now they put you here on this balcony where you have to look down. The security itself don’t know the alternative entrances are for PWDs. So now you sit there whilst everyone is in a hurry and you are waiting for someone from another side to send messages of where I should go in.’ (Dylan, student, theatre attendee, FG3, March 2017:27)

‘I think most of them [*youth with disabilities*] wouldn’t attend things when there was something but I’m always there, because there’s always a venue with the stairs, a venue with the smaller door, so they [*youth with disabilities*] can’t be accommodated.’ (Chidera, student, theatre attendee, FG3, 2017:36)

### Striving to fit in

Some challenges were specific to certain groups of participants. The learners from the High School for the Deaf and the Tertiary Institute for the Deaf struggled with the anxiety of having to engage with hearing people, within systems and structures that are more exclusive rather than inclusive of them. Although some deaf people have acquired the skills to interact in a world more attuned to hearing people, the anxiety also occurred at a personal level:

‘When we are now outside, working and done with our education, how are we going to communicate with the others, with the hearing?’ (Brandon, student, theatre attendee, FG1, 2017)

‘Some deaf people can mix with hearing people and then some deaf people do not know the skills and techniques required to communicate with hearing people. So some deaf people can do it, they can do it, but I just do not know how to communicate with the hearing.’ (Eric, student, theatre attendee, FG2, March 2017)

Despite these highlighted challenges, participants believe that inclusive structures and systems as well as their deeply held aspirations are necessary for successful inclusion. The need to prove themselves despite these challenges is reflected in the following statement:

‘No, I think that one is a person showing the world that even if I am disabled, let me show them that I can do it, you can do it.’ (Brandon, student, theatre attendee, FG1, 2017:21)

‘Now I can showcase my hobby to a lot of people, and that will also contribute into changing the mindsets of the people who think that disability is also in the head, because when they see you they like, “Agh, you stupid, you know, you can’t think” … I’m going to showcase the work that I’m doing, my hobby, because designing is a hobby for me; I’m not a professional designer, I’ve never been to designing school, but I love fashion. And people should see that we can also dress up, like make up, like girly stuff, and we like the neatness, you know. It’s not just about, agh, we dressed because we have to get dressed, you know? There is more to us than what meets the eye!’ (Chidera, student, theatre attendee, FG3, 2017:38)

The given section expands on the outcomes of the study, further synthesising the key findings above to highlight the key tenets of an inclusive framework from the participant narratives above.

## Discussion

‘People will always act different to what they don’t know, because now I understand why able bodies don’t really consider PWD, because they just seem like, “Agh, you know, that person, I don’t really know what they are about.” It’s because they don’t understand you, but if a person understands you and has a better understanding of who you are, then they will be more open to what is happening around them … so that people know that we are there, we are not going anywhere, we are not something that’s gonna vanish into thin air anytime soon, so they must get used to us and get to know us.’ (Chidera, student, theatre attendee, FG3, March 2017:37)

The given quote by one of the youths with disabilities reflects the general sentiment expressed by the study participants. In consideration of a more disability-inclusive framework that consciously takes note of context and holistic engagement of the youth with disabilities within society, there are certain key factors that have emerged from participant narratives that would contribute positively to support a contextually relevant and appropriate disability-inclusive framework within the South African context. These factors can be synthesised into three main areas discussed here.

### Enhancement of personal capacity, agency and skills development for youth with disabilities

There is a worldwide crisis of youth unemployment, including in South Africa. Critical to this is the social isolation and marginalisation of youth with disabilities, who consist of a staggering 71% of people who are unemployed in South Africa (Statistics South Africa 2011). The South African government identified youth with disabilities as a vulnerable group and a target population for redressal more than a decade ago, but they still do not constitute a government priority (Engelbrecht, Shaw & Van Niekerk 2017).

In a study that explored the availability of work transition programmes for youth with disabilities in South Africa, authors identified inadequate research that focuses on this group, with little data disaggregated to highlight information on their status. These include the supportive and inclusive structures needed to enable them to realise their aspirations (Engelbrecht et al. 2017). Even more critical is the need to hear from the youth themselves, in support of their agency and autonomy. Research that involves youth with disabilities, which elicits their narratives, is key to challenging societal normative assumptions that impact negatively on the creation of inclusive structures to integrate youth with disabilities into all aspects of society (Teachman et al. 2018). This supports the given quote by a participant who insists that one of their biggest challenges is that society is largely unfamiliar with and distanced from the realities of youth with disabilities. This can be addressed through focused research to elicit their narratives.

### Supporting and empowering families to inform sustainable change

Disability is not only a physiological, societal and systemic issue but also a deeply personal and emotive issue. Participants in this study speak about a personal, internal resilience (Daly 2020) that exists, making them strive to learn new skills to sustain themselves despite all odds (Le Roux 2018). Families are key support systems for resilience (Ohajunwa 2019) and participants often referred to the role their families or parents played in their lives, helping them to become resilient. The community-based rehabilitation framework (WHO 2010) recognises the key and pivotal role which families play when it comes to the inclusion of PWD. Community Based Rehabilitation (CBR) advocates for the ongoing inclusion of families and communities in programmes planned for PWD as part of an inclusive framework. Although they battle fear, anxiety, social stereotyping (Shah et al. 2015) and oftentimes a lack of self-esteem and confidence related to social interactions (Max-Neef 2009), due to long-term stigmatisation (McLaughlin, Bell & Stringer 2004) and abuse that they experience, there is a resolve to keep aspiring, supported by their families (Le Roux 2018). Daly (2020) conducted an integrated literature review of resilience in the fields of psychology, sociology, philosophy, education and nursing. One of the key outcomes of the study is that closeness to resources is vital to inclusion and builds resilient wellbeing for youth with disabilities. The family is one such resource, which must be utilized through empowering families as a tool for inclusion of youth with disabilities.

### Effecting systemic and structural changes for inclusion of youth with disabilities

One of the needs identified by youth with disabilities is the relevance of systemic and structural accommodation of their needs. Closely linked to this is the importance of general disability sensitivity or awareness to support societal inclusion and access to opportunities. Disability awareness would speak to negative attitudes suffered by youth with disabilities, even within inclusive spaces, as described by a participant here.

Disability is a multidimensional concept, and the lived experience of it is influenced by systematic differences based on gender, disability, race or ethnicity and socio-economic status (Shogren & Shaw 2016). The historical imbalances imposed by South Africa’s past constitute certain barriers for these young people. Youth with disabilities from historically disadvantaged communities perceive their white counterparts as having more access to resources that facilitate their inclusion into society than they do (Le Roux 2018; Roberts 2004). This is especially seen in the area of accessible transportation, which is key to accessing opportunities for schooling, employment, recreation, places of worship and many other spaces of advancement.

An inclusive disability framework must cater for all areas of youth development – physical, cognitive, social, emotional well-being and learning (Lee & Ho 2018). A holistic approach that accounts for all aspects of their humanity (Ohajunwa, Mji & Chimbala-Kalenga 2021) is key to the development of self-determination, which is a significant predictor of post-school success for youth with disabilities (Shogren & Shaw 2016). One sees the impact of the legacy and history of South Africa’s apartheid on the self-determination and aspirations of youth with disabilities who come from communities that were subjugated during this era. Redress is an important factor to inform the inclusion of youth with disabilities. There is a need to recognise that all youth with disabilities are not equal in terms of resources to access opportunities and their sense of self. Therefore, systems must be set up to further assist youth with disabilities from disadvantaged communities to be able to dream and achieve. Daly (2020) agrees with the summation above, stating that proximity to resources is key to accessing opportunities and building resilience of the youth with disabilities to facilitate inclusion.

Related to a sense of self, Meiring (2015:5) focuses on embodiment within the southern African context. He emphasises the importance of taking cognisance of the bodily experiences of black people in South Africa and having a deep sensitivity for the profound impact of colonialism, apartheid and even post-apartheid on the black body. This is applicable to youth with disabilities, and there should therefore be a conscious move to address this deficit within an inclusive framework.

Meiring (2016) discussed the notion of ‘embodied sensing’, stating that all people have the challenge of sometimes being unable to make meaning of their worlds with words: we all embody this sense of meaning, even when we cannot explain it with words. One challenge is that PWDs are often told by able-bodied people who they are and what to believe about themselves. Meanwhile, even if they cannot speak, or are hesitant to speak, youth with disabilities are much more than their disabled bodies; they also have this embodied sense of themselves and should be given the right to define for themselves who they want to be. Harnessing this sense of embodiment to inform the inclusion of youth with disabilities is critical. It will entail creating unhurried spaces of dialogue and authentic, nonthreatening engagement that allows for self-expression. As this study has shown, Artscape and NGOs who bring youth with disabilities to interact and attend performances can provide a space for these facilitated dialogues.

## Conclusion

Globally and in South Africa, youth with disabilities are not prioritised as they should be by governments. Although youth constitute most of the working population in South Africa, they still experience marginalisation and minimal access to opportunities at systemic and personal levels, in part because of South Africa’s past injustices and a general lack of disability sensitivity within society. From the narratives of inclusion that emanated from youth with disabilities in this study, three main factors that contribute to an inclusive framework have been identified. They are enhancement of personal capacity, agency and skills development for youth with disabilities, supporting and empowering families to inform sustainable change and effecting systemic and structural changes for the inclusion of youth with disabilities.

## Study implications

Finally, based on the research presented, this article makes the following recommendations:

- Promotion of societal systems and structures that actively facilitate the inclusion of youth with disabilities. Accessibility to all public spaces should be ensured, including places of social interaction, to facilitate inclusion.
- Adopt inclusive practices at all levels of society, with government, academia and NGOs providing disability sensitivity programmes and training, so that even when inclusive structures are built, managers of these structures are aware of the need for further accommodation as required.
- The experience of youth with disabilities from disadvantaged communities specifically, is informed by race, gender, socio-economic and spatial divisions of an apartheid legacy. Therefore, in the interest of equity, additional government funding and training must be made available to support these youth and their families to access equal opportunities at par with their peers in order to facilitate inclusion.
